# Association of Robotic Assistance With Short-term Outcomes After Coronary Artery Bypass Grafting

**DOI:** 10.1016/j.atssr.2025.03.007

**Published:** 2025-03-20

**Authors:** Arjun Verma, Justin J. Kim, Sara Sakowitz, Yas Sanaiha, Joseph Hadaya, Peyman Benharash

**Affiliations:** 1 Center for Advanced Surgical and Interventional Technology, Department of Surgery, David Geffen School of Medicine at UCLA, University of California, Los Angeles, Los Angeles, California; 2 Harvard Medical School, Boston, Massachusetts; 3 Division of Cardiac Surgery, Department of Surgery, David Geffen School of Medicine at UCLA, University of California, Los Angeles, Los Angeles, California

## Abstract

**Background:**

Coronary artery bypass grafting (CABG) is traditionally performed though median sternotomy for multivessel coronary artery disease. Robotic CABG, a viable alternative, comprises less than 1% of CABG procedures in the United States despite its potential benefits. This study aimed to compare the trends and outcomes of conventional and robotic CABG by using a contemporary national cohort.

**Methods:**

A retrospective study was conducted using the 2016 to 2020 Nationwide Readmissions Database (NRD). Adult patients (aged ≥18 years) who underwent single-vessel CABG were identified using International Classification of Diseases, 10th revision procedure codes. Patients were categorized into robotic (totally endoscopic or robotic-assisted) and conventional CABG cohorts. Outcomes evaluated included in-hospital mortality, major adverse events (MAEs), length of stay, hospitalization costs, nonhome discharge, and 30-day readmissions.

**Results:**

Among 21,870 patients, 3433 (15.7%) underwent robotic CABG. The use of robotic CABG increased modestly over the study period. Patients who underwent robotic CABG had lower in-hospital mortality (0.4% vs 1.7%; *P* < .001) and MAEs (11.4% vs 18.9%; *P* < .001) compared with conventional CABG. Moreover, the robotic CABG cohort was associated with shorter length of stay and reduced hospitalization costs. After adjusting for baseline characteristics, robotic CABG showed lower odds of in-hospital mortality (adjusted odds ratio, 0.35; 95% CI, 0.15-0.84; *P* = .019) and MAEs (adjusted odds ratio, 0.72; 95% CI, 0.59-0.88; *P* = .001).

**Conclusions:**

Robotic CABG is associated with reduced in-hospital mortality, complications, LOS, and hospitalization costs compared with conventional CABG. Despite these benefits, its adoption remains limited, potentially because of the steep learning curve and resource requirements. Further efforts to overcome these barriers could enhance the adoption of robotic CABG and improve patient outcomes.

**In Short**

▪ Robotic CABG showed significantly better outcomes compared with conventional CABG, with lower in-hospital mortality, fewer complications, shorter hospital stays, and reduced costs by approximately $3000 per case.
▪ Despite these advantages, robotic CABG remains underused, accounting for less than 1% of CABG procedures in the United States, with minimal growth in adoption among hospitals (stagnant at 8.9%) because of steep learning curves and resource requirements.

Coronary artery bypass grafting (CABG) remains the gold standard treatment for multivessel coronary artery disease.^1^ Although this operation is traditionally performed through median sternotomy, technologic advances have allowed robotic-assisted CABG to emerge as a viable alternative to conventional single-vessel CABG or as a hybrid adjunct to percutaneous coronary intervention (PCI).^2^ Nonetheless, <1% of coronary artery bypass grafting procedures in the United States are currently performed with robotic assistance.^3^

The slow adoption of robotic CABG may, in part, be attributable to the steep learning curve and extensive resources required to initiate a robotic program.^4^ Moreover, existing analyses comparing outcomes of robotic and conventional CABG have yielded conflicting results.^2^ Therefore, the present national study analyzed a contemporary cohort of patients undergoing single-vessel coronary revascularization and examined trends and outcomes of conventional and robotic CABG. We hypothesized robotic assistance to be associated with reduced mortality and complications but increased resource use, relative to conventional CABG.

## Material and Methods

All elective adult hospitalizations entailing single-vessel CABG were identified within the 2016 to 2020 Nationwide Readmissions Database (NRD) (Supplemental Table 1).^5^ Patients undergoing concomitant cardiac operations were not considered. Records missing data for age, sex, mortality, length of stay (LOS), or costs were excluded (<1%). Patients undergoing totally endoscopic (distal anastomosis performed endoscopically) or robotic-assisted CABG (distal anastomosis performed through minithoracotomy) were tabulated using relevant International Classification of Diseases, 10th revision (ICD-10) procedure codes and grouped as the robotic cohort. Those undergoing open CABG comprised the conventional cohort.

Demographic and hospital characteristics were defined in accordance with the NRD Data Dictionary.^6^ The van Walraven modification of the Elixhauser Comorbidity Index was used to quantify the burden of chronic conditions. Individual comorbidities, use of cardiopulmonary bypass, and PCI during the index hospitalization were tabulated using ICD-10 diagnosis and procedure codes. Stroke, cardiac complications (ventricular tachycardia or fibrillation, tamponade, cardiac arrest), venous thromboembolism, respiratory failure, pneumonia, sepsis, renal failure, and reoperation were similarly defined and grouped as major adverse events (MAEs). Hospitalization costs were calculated by application of hospital-specific cost-to-charge ratios and inflation adjusted to the 2020 Personal Health Index.

Temporal trends were assessed using a nonparametric, rank-based test. Categorical variables are reported as percentages and are compared using the Pearson χ^2^ statistic. Normally distributed continuous variables are reported as means (SD) and are compared using the adjusted Wald test. Continuous variables with skewed distributions are shown as medians with interquartile range (IQR) and are compared using the Mann-Whitney *U* test. Entropy balancing was used to adjust for inherent differences between the conventional and robotic cohorts. After entropy balancing, multivariable linear and logistic regressions were developed to assess the risk-adjusted association of operative approach with outcomes of interest. Regression outputs are reported as adjusted odds ratios (AORs) or beta coefficients (β) with 95% CIs. Statistical significance was set at α = .05. All analyses were performed using Stata software version 16.1 (StataCorp). This study was deemed exempt from full review by the Institutional Review Board at the University of California, Los Angeles.

## Results

**Figure 1:**
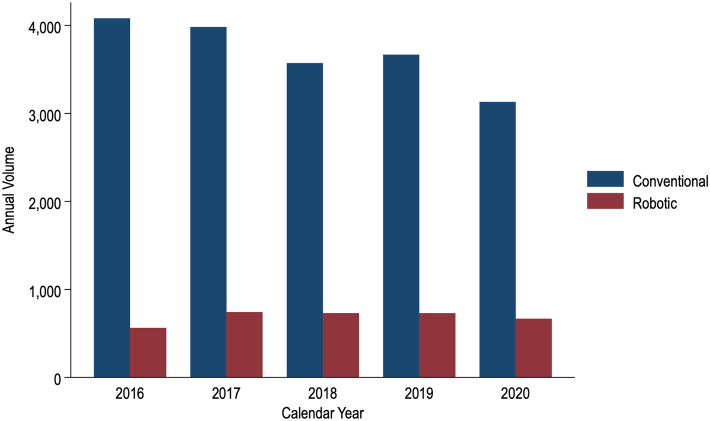
Trends in the use of conventional and robotic-assisted single-vessel coronary artery bypass grafting in the United States.

Of an estimated 21,870 patients meeting study criteria, 3433 (15.7%) comprised the robotic cohort, and 18,437 (84.3%) represented the conventional cohort. Within the robotic cohort, 18.4% underwent totally endoscopic CABG. Patient characteristics between totally endoscopic and robotic-assisted minimally invasive CABG, both subsets of the robotic cohort, were comparable (Supplemental Table 2). National use of robotic CABG increased modestly over the study period, from 563 patients in 2016 to 667 in 2020 (nptrend < .001) (Figure 1). However, the proportion of hospitals using robotic CABG did not change (8.9%; nptrend = .40).

Compared with the conventional group, patients in the robotic group had a similar distribution of age (65.0 ± 10.5 years vs 65.5 ± 11.4 years; *P* = .12) but were less commonly female (30.0% vs 20.8%; *P* < .001) and had a lower burden of comorbidities as measured by the Elixhauser Comorbidity Index (4.1 ± 2.0 years vs 3.5 ± 2.0 years; *P* < .001). Specifically, peripheral vascular disease, neurologic disorders, and coagulopathy were less prevalent among the robotic cohort (Table 1). Of note, the use of cardiopulmonary bypass was significantly lower within the robotic group (5.9% vs 53.4%; *P* < .001). Internal mammary arteries were more frequently used in the robotic cohort, compared with the conventional cohort (98.6% vs 74.8%; *P* < .001). Approximately 805 patients underwent PCI during the index hospitalization, with a higher incidence in the robotic group (8.8% vs 2.9%; *P* < .001). Among patients who underwent PCI, same-day PCI was more frequent among conventional CABG patients (371 of 805; 73.9%), whereas patients those undergoing robotic CABG more commonly had PCI ≥1 day after surgery (92.6%; *P* < .001).

**Table 1:** Comparison of Patients Undergoing Conventional and Robotic-assisted Coronary Artery Bypass Grafting

Variable	Conventional	Robotic	*P* Value
	n = 18,437	n = 3433	
Age, y	65.0 (10.5)	65.5 (11.4)	.12
Female sex	30.0	20.8	<.001
Cardiopulmonary bypass	53.4	5.9	<.001
Percutaneous coronary intervention	2.9	8.8	<.001
Elixhauser Comorbidity Score	4.1 (2.0)	3.5 (2.0)	<.001
Congestive heart failure	27.5	24.3	.09
Valve disease	18.4	11.5	<.001
Pulmonary circulation disorder	4.6	2.7	.005
Peripheral vascular disease	20.3	10.0	<.001
Other neurologic disorder	5.4	2.8	<.001
Chronic pulmonary disease	23.3	19.6	.005
Liver disease	3.0	2.4	.24
Coagulopathy	18.5	10.4	<.001
Obesity	29.5	24.5	<.001
Income quartile			<.001
76th-100th	17.1	29.9	
51st-75th	25.2	32.1	
26th-50th	28.7	22.2	
1st-25th	29.0	15.8	
Primary payer			.11
Private	33.4	37.2	
Medicare	56.5	53.5	
Medicaid	6.6	6.6	
Other	3.5	2.7	
Hospital location/teaching status			<.001
Rural	3.3	0.3	
Metropolitan nonteaching	15.1	5.1	
Metropolitan teaching	81.6	94.6	

Values are mean (SD) or %.

Patients undergoing robotic CABG faced lower in-hospital mortality and MAEs, compared to patients undergoing conventional CABG. Although the need for reoperation was similar between the groups, the conventional cohort had higher incidences of cardiac complications, respiratory failure, and sepsis relative to those in the robotic group (Table 2). Moreover, the robotic cohort experienced shorter LOS and accrued lower hospitalization costs. Patients undergoing robotic CABG faced reduced rates of nonhome discharge, but unaltered 30-day nonelective readmission, compared with patients undergoing conventional CABG.

**Table 2:** Comparison of Outcomes Between Patients Undergoing Conventional and Robotic-assisted Coronary Artery Bypass Grafting

Variable	Conventional	Robotic	*P* Value
	n = 18,437	n = 3433	
In-hospital mortality	1.7	0.4	<.001
Major adverse event	18.9	11.4	<.001
Reoperation	1.0	1.4	.22
Sepsis	1.5	0.3	<.001
Renal failure	0.5	a	.07
Respiratory failure	11.6	6.8	<.001
Pneumonia	3.3	1.5	.001
Thromboembolism	1.2	0.3	.003
Cardiac complication	6.4	2.9	<.001
Stroke	1.7	0.8	.004
Length of stay, d	5 (4-7)	4 (3-5)	<.001
Index hospitalization costs, $1000s	32.9 (24.9-45.7)	27.2 (21.4-37.4)	<.001
Nonhome discharge	12.4	5.0	<.001
30-day nonelective readmission	8.4	7.9	.48

Values are % or median (interquartile range).

a Indicates n <10.

**Figure 2:**
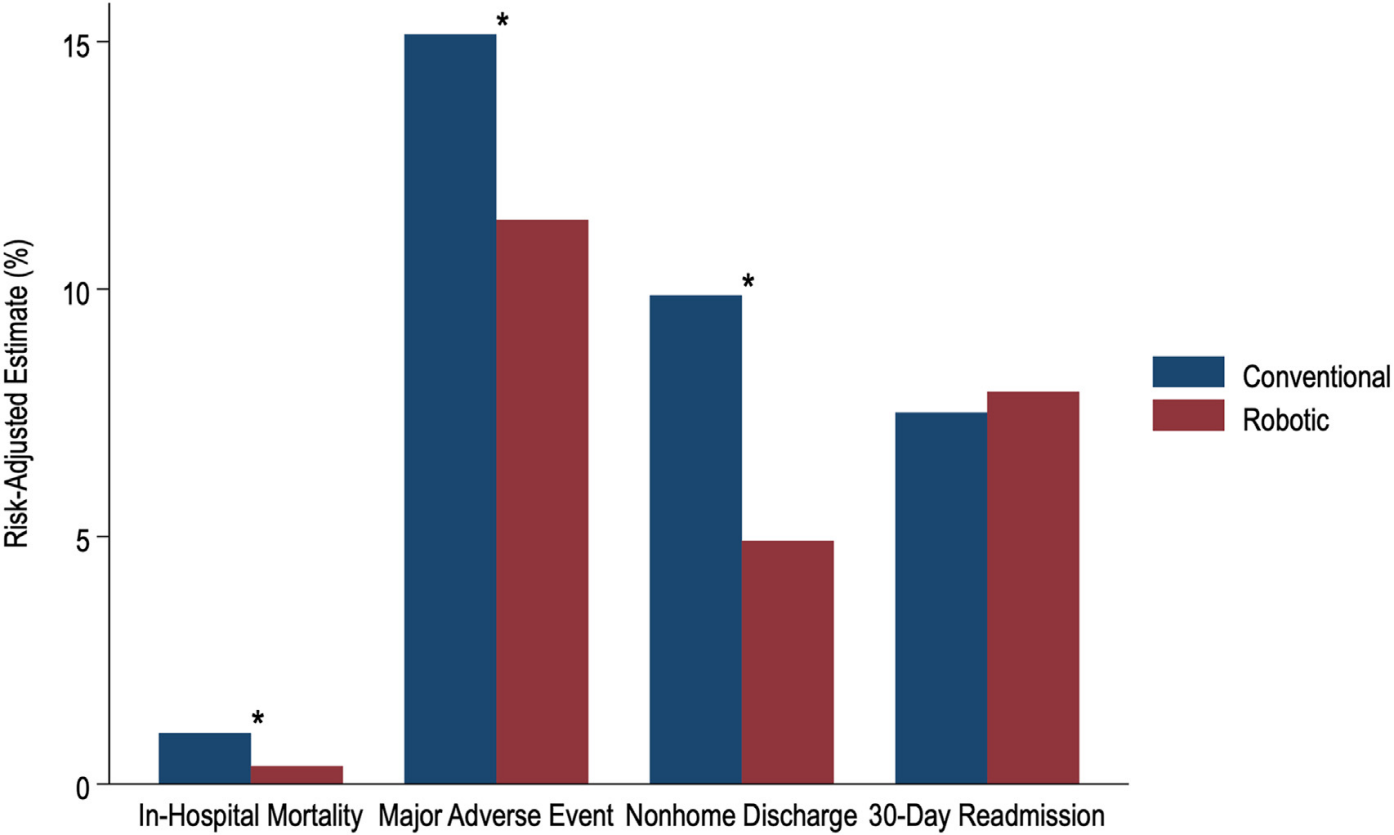
Risk-adjusted association of robotic-assisted coronary artery bypass grafting with clinical end points, relative to conventional. Major adverse events were defined as a composite of stroke, cardiac complications (ventricular tachycardia or fibrillation, tamponade, cardiac arrest), venous thromboembolism, respiratory failure, pneumonia, sepsis, renal failure, and reoperation. ∗*P* < .05.

As shown in Supplemental Table 3, entropy balancing resulted in comparable distributions of all baseline characteristics between the conventional and robotic cohorts. After risk -adjustment, robotic CABG was associated with significantly reduced odds of in-hospital mortality (AOR, 0.35; 95% CI, 0.15-0.84; *P* = .019) and MAEs (AOR, 0.72; 95% CI, 0.59-0.88; *P* = .001), with conventional CABG as reference. Robotic CABG was linked to a 0.9-day decrement in LOS (95% CI, −1.0 to −0.7; *P* < .001) and a $3000 reduction in hospitalization costs (95% CI, −5000 to −1000; *P* = .001). Relative to the conventional cohort, the robotic group further demonstrated lower odds of nonhome discharge (*P* < .001) but an equivalent likelihood of unplanned 30-day readmission (*P* = .59) (Figure 2).

## Comment

The present work used a national database to evaluate trends and outcomes of single-vessel robotic CABG. Over the 5-year study period, a 20% increase in nationwide robotic CABG volume was noted, although the proportion of centers using this approach remained stagnant. We further found that patients undergoing robotic CABG faced significantly reduced risk-adjusted odds of in-hospital mortality and complications, relative to conventional CABG. Importantly, patients who underwent robotic CABG experienced shorter lengths of stay and accrued lower hospitalization costs.

Congruent with our findings, several previously published studies have highlighted the markedly slow adoption of robotic CABG in the United States.^7^ Multiple factors may contribute to limited adoption of this modality at the provider and center level. First, for patients requiring single-vessel revascularization, PCI represents a suitable alternative to robotic CABG and may be foregone only for anatomic considerations. Furthermore, existing studies examining the impact of surgical approach on long-term graft patency are limited by sample size and follow up duration.^8^ Finally, initiation of a robotic program requires significant financial and professional investment, related to both the cost of the robotic surgery platform and steep learning curve.^4^ The observations of the present work indicate that such barriers continue to hinder the adoption of robotic assistance in coronary surgery. These obstacles may be overcome through multidisciplinary initiatives between industry and clinical stakeholders that aim to curb short- and long-term costs and flatten the learning curve.

Given that resource use is a potential driver of hesitancy in the adoption of robotic surgery, we assessed overall LOS and total hospitalization costs within our national cohort of robotic CABG patients. The robotic approach was associated with a 1-day reduction in LOS and a $3000 decrement in hospitalization costs, relative to conventional. The reasons underlying this observation are multifactorial but may relate to the reduced incidence of costly complications and the opportunity for early ambulation. Yokoyama and colleagues^9^ examined the 2012 to 2017 National Inpatient Sample and noted a ∼$5000 reduction in costs associated with robotic CABG. The present study adds to these findings by using a contemporary data set, examining a more homogenous cohort comprising single-vessel CABG, and using entropy balancing as a robust risk adjustment method.^10^ Taken together, our findings suggest that robotic CABG is more cost effective at the patient level.

This study has several important limitations. First, given the retrospective nature of the study design, we were unable to establish causal relationships between operative approach and outcomes of interest. Furthermore, the NRD does not contain granular clinical information, including operative or cardiopulmonary bypass times, laboratory tests, and imaging studies. Importantly, data concerning graft patency as well as the incidence of midterm or long-term coronary reintervention are not available in the NRD. Within the data source, costs are reported as aggregates and are predicted to include direct and indirect expenses related to the hospitalization. Although we used entropy balancing and multivariable regression to adjust for differences in baseline characteristics between cohorts, the influence of selection bias on the present findings cannot be ignored.

In conclusion, the present study examined a national cohort of patients undergoing single-vessel robotic CABG and found reduced mortality, complications, and resource use to be associated with this approach, relative to conventional CABG. Our findings demonstrate that robotic CABG has acceptable short-term clinical outcomes and may provide a financial advantage to adopting institutions.
