# Supplementary material for: Association of Robotic Assistance With Short-term Outcomes After Coronary Artery Bypass Grafting
Source: Ann Thorac Surg Short Rep. 2025 Mar 20;3(3):603–8. doi: 10.1016/j.atssr.2025.03.007 (PMC12559596; doi:10.1016/j.atssr.2025.03.007)
Supplement: Supplementary Table 1 [file mmc1.docx]

Supplemental Table 1: *International Classification of Diseases 10^th^ Revision (ICD-10) procedure codes used to identify the study cohort. CABG: Coronary Artery Bypass Grafting.*

| **Procedure** | **ICD-10 Code** |
| --- | --- |
| Conventional Single-Vessel CABG | 02100, 02110, 02120, 02130 |
| Totally Endoscopic Single-Vessel CABG | 02104, 02114, 02124, 02134  AND  8E0W0CZ, 8E0W4CZ |
| Robotic-Assisted Single-Vessel CABG | 02104, 02114, 02124, 02134  OR  02100, 02110, 02120, 02130  AND  8E0W0CZ, 8E0W4CZ |
