# Supplementary material for: Association of Robotic Assistance With Short-term Outcomes After Coronary Artery Bypass Grafting
Source: Ann Thorac Surg Short Rep. 2025 Mar 20;3(3):603–8. doi: 10.1016/j.atssr.2025.03.007 (PMC12559596; doi:10.1016/j.atssr.2025.03.007)
Supplement: Supplementary Table 2 [file mmc2.docx]

Supplemental Table 2: Comparison of totally endoscopic and robotic-assisted coronary artery bypass grafting patients.

| **Parameter** | **Robotic-Assisted** | **Totally Endoscopic** | **P-Value** |
| --- | --- | --- | --- |
|  | **n=2,801** | **n=632** |  |
| Age (years, mean±SD) | 65.6±10.6 | 65.5±9.9 | 0.88 |
| Female Sex (%) | 20.6 | 21.7 | 0.62 |
|  |  |  |  |
| Cardiopulmonary Bypass (%) | 6.7 | 2.4 | 0.004 |
| Percutaneous Coronary Intervention (%) | 8.5 | 10.2 | 0.26 |
|  |  |  |  |
| Elixhauser Comorbidity Score (mean±SD) | 3.6±1.9 | 3.4±1.8 | 0.19 |
| Congestive Heart Failure (%) | 24.6 | 23.1 | 0.55 |
| Valve Disease (%) | 11.6 | 11.2 | 0.84 |
| Pulmonary Circulation Disorder (%) | 0.29 | 1.9 | 0.32 |
| Peripheral Vascular Disease (%) | 10.1 | 9.7 | 0.81 |
| Other Neurologic Disorder (%) | 2.7 | 3.2 | 0.57 |
| Chronic Pulmonary Disease (%) | 19.8 | 18.5 | 0.56 |
| Liver Disease (%) | 2.3 | 2.8 | 0.53 |
| Coagulopathy (%) | 10.5 | 9.9 | 0.73 |
| Obesity (%) | 24.5 | 24.7 | 0.90 |
|  |  |  |  |
| Income Quartile (%) | |  | 0.25 |
| 76th-100th | 29.8 | 30.0 |  |
| 51st-75th | 33.2 | 27.3 |  |
| 26th-50th | 21.4 | 25.4 |  |
| 1st-25th | 15.5 | 17.2 |  |
|  |  |  |  |
| Primary Payer (%) | |  | 0.027 |
| Private | 37.7 | 34.7 |  |
| Medicare | 53.7 | 52.4 |  |
| Medicaid | 6.4 | 7.6 |  |
| Other | 2.2 | 5.2 |  |
|  |  |  |  |
| Hospital Location/Teaching Status (%) | | | 0.16 |
| Rural | * | * |  |
| Metropolitan Non-Teaching | 5.5 | 3.2 |  |
| Metropolitan Teaching | 94.2 | 96.3 |  |
