# Supplementary material for: Association of Robotic Assistance With Short-term Outcomes After Coronary Artery Bypass Grafting
Source: Ann Thorac Surg Short Rep. 2025 Mar 20;3(3):603–8. doi: 10.1016/j.atssr.2025.03.007 (PMC12559596; doi:10.1016/j.atssr.2025.03.007)
Supplement: Supplementary Table 3 [file mmc3.docx]

Supplemental Table 3: Comparison of conventional and robotic-assisted coronary artery bypass grafting patients after entropy balancing.

| **Parameter** | **Conventional** | **Robotic** | **P-Value** |
| --- | --- | --- | --- |
|  | **n=4,054** | **n=3,381** |  |
| Age (years, mean±SD) | 65.7±12.7 | 65.5±6.7 | 0.64 |
| Female Sex (%) | 20.7 | 20.9 | 0.83 |
|  |  |  |  |
| Cardiopulmonary Bypass (%) | 6.0 | 5.9 | 0.92 |
| Percutaneous Coronary Intervention (%) | 8.7 | 8.8 | 0.92 |
|  |  |  |  |
| Elixhauser Comorbidity Score (mean±SD) | 3.5±2.3 | 3.5±1.2 | 0.91 |
| Congestive Heart Failure (%) | 23.8 | 24.1 | 0.15 |
| Valve Disease (%) | 11.4 | 11.5 | 0.96 |
| Pulmonary Circulation Disorder (%) | 2.5 | 2.7 | 0.69 |
| Peripheral Vascular Disease (%) | 10.1 | 10.0 | 0.92 |
| Other Neurologic Disorder (%) | 2.7 | 2.9 | 0.25 |
| Chronic Pulmonary Disease (%) | 20.9 | 19.6 | 0.4 |
| Liver Disease (%) | 2.2 | 2.4 | 0.59 |
| Coagulopathy (%) | 9.1 | 10.5 | 0.29 |
| Obesity (%) | 25.3 | 24.5 | 0.66 |
|  |  |  |  |
| Income Quartile (%) | |  | 0.84 |
| 76th-100th | 28.2 | 29.9 |  |
| 51st-75th | 33.7 | 32.2 |  |
| 26th-50th | 22.2 | 22.1 |  |
| 1st-25th | 15.9 | 15.9 |  |
|  |  |  |  |
| Primary Payer (%) | |  | 0.77 |
| Private | 36.6 | 37.3 |  |
| Medicare | 54.4 | 53.3 |  |
| Medicaid | 6.7 | 6.7 |  |
| Other | 2.3 | 2.8 |  |
|  |  |  |  |
| Hospital Location/Teaching Status (%) | | | 0.92 |
| Rural | 0.2 | 0.3 |  |
| Metropolitan Non-Teaching | 4.7 | 4.8 |  |
| Metropolitan Teaching | 95.1 | 94.8 |  |
